# Left double polar renal arteries, left triplicate (preaortic, accessory and retroaortic) renal veins associated with extrinsic pelviureteric junction obstruction and posterior nutcracker phenomenon

**DOI:** 10.1259/bjrcr.20200086

**Published:** 2021-05-14

**Authors:** Martin Ian Kamanda

**Affiliations:** 1 HSM University of Nairobi, University of Johannesburg, Johannesburg, South Africa; 2 M.P.Shah Hospital, Nairobi, Kenya

## Abstract

The renal vasculature and its various congenital anomalies have been studied and documented widely in the literature. However, the concomitant occurrence of renovascular morphological anomalies with vascular compression phenomena in a single patient is a rarity. This is a case of a patient with double left renal arteries, preaortic, accessory and retroaortic left renal veins. There was also associated with vascular compression phenomena in the form of posterior nutcracker phenomenon and pelviureteric junction obstruction (PUJ) due to the double-crossing inferior left polar renal artery and retroaortic vein.

## Clinical presentation

A 24-year-old was sent for a CT pyelogram with a history of persistent left flank pain before surgical management. Previous CT pyelogram done a year earlier in another institution showed left PUJ obstruction. On the current non-contrast scan, there was moderate hydronephrosis (Grade III) with PUJ obstruction due to a suspected lower pole crossing accessory renal artery. The protocol was modified to CT renal angiogram/venogram to evaluate the renal vasculature and subsequent aetiology of the PUJ obstruction.

## Imaging protocol

The patient was scanned using a Somatom Definition AS, 128 slice, Siemens, Elargen, Germany. The patient’s urea and creatinine levels were normal. The patient signed an informed consent authorizing the use of intravenous contrast media. He was also instructed to take water with the aim of filling the bladder.

An i.v. access line (right median cubital vein) was identified and a 20-gauge (pink) cannula was inserted 80 mls of Ultravist 370 at the rate of 4 mls/s was administered using a pump injector. The early arterial, venous and delayed scans were acquired 15 s, 23 s and 8 min, respectively, after bolus tracking. For the early arterial and venous phase, the scan area was limited to the kidneys. A delayed excretory scan after 24 h was done to demonstrate the PUJ stenosis and distal ureteric anatomy.

Multiplanar reconstructions of slice 2 mm with a reconstruction interval of 2 mm (B20 smooth kernel, abdomen window) were done. A slice thickness of 0.75 mm and increment of 0.3 mm (B10 smooth kernel, abdomen window) were used for maximum intensity projections and volume rendered technique.

## Ct findings

**Figure 1.**
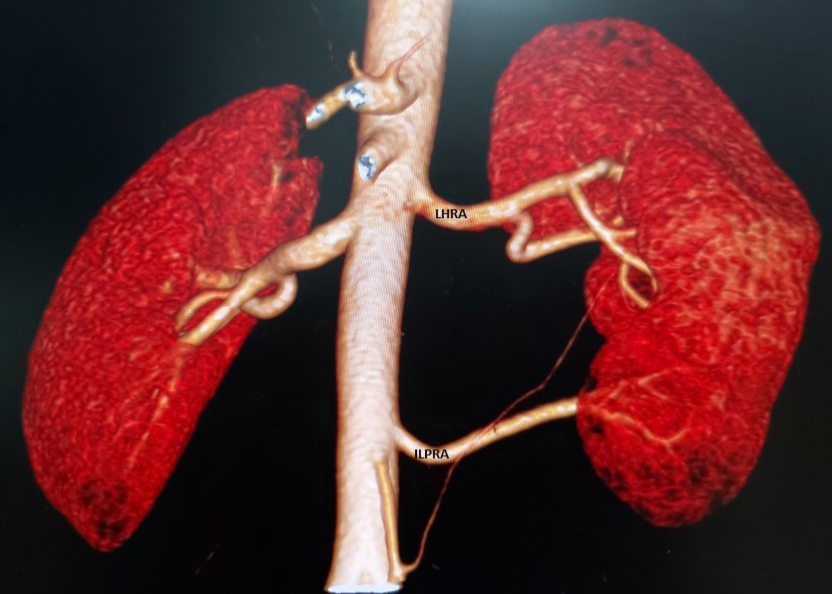
VRT image showing the Left Hilar Renal Artery (LHRA) and Inferior Left Polar Renal Artery (ILPRA).

The right kidney, right ureter and bladder were normal. There was also no evidence of renal hypertension, varices or thrombosis. However, the left kidney had a complex renovascular morphology with associated posterior nutcracker phenomenon and PUJ obstruction. The left hilar renal artery (LHRA) had a nearly horizontal course from its origin from the aorta and divided into three presegmental branches (Figure 1). The inferior left polar renal artery (ILPRA) had an intimate and parallel course with the retroaortic left renal vein (RLRV) laterally. The ILPRA divided into two presegmental branches (Figure 1).

**Figure 2.**
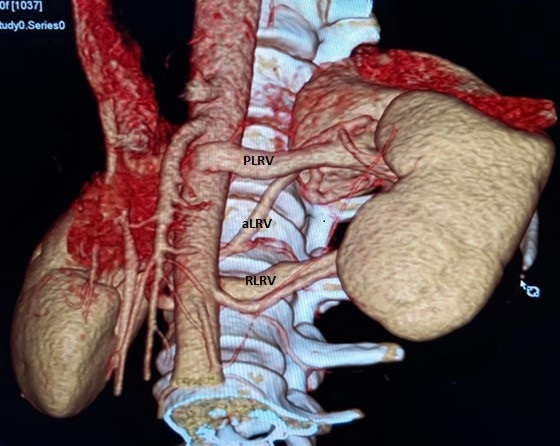
Anterior view of a VRT image depicting the Preaortic Left Renal Vein (PLRV), accessory Left Renal Vein(aLRV) and Retroaortic Left Renal Vein (RLRV).

**Figure 3.**
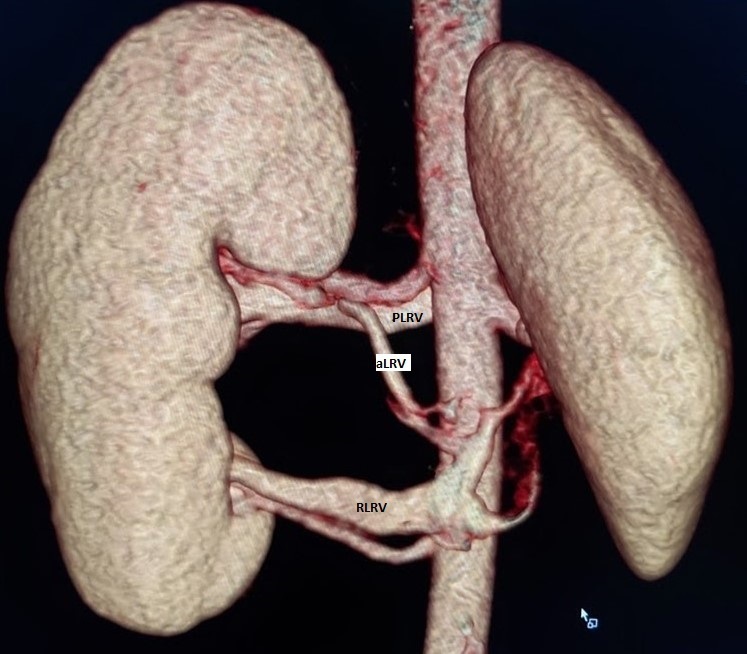
Posterior view of a VRT image depicting the Preaortic Left Renal Vein (PLRV), accessory Left renal vein(aLRV) and Retroaortic Left Renal Vein (RLRV).

The left renal venous morphology comprised of superior pole preaortic left renal vein (PLRV) and inferior pole retroaortic left renal vein (RLRV) and middle pole accessory left renal vein (aLRV) (Figures 2 and 3). The PLRV draining the upper pole had a horizontal course and posterior to the LHRA joining the inferior vena cava (IVC) at the L1 level. The diameter of the proximal portion of this vein narrowed down between the aorta and the superior mesenteric artery giving a CT impression of the beak sign. The beak angle was measured to be greater than 38 degrees, which was within normal limits. The diameter of the PLRV at hilar and aortomesenteric regions were 0.76 cm and 0.28 cm, respectively, which gave a ratio of 2.7 which were within normal limits.

The middle pole aLRV had an oblique course in the craniocaudal direction to join the RLRV just has it joins the IVC (Figures 2 and 3). Proximally, this vein had been displaced superiorly by the dilated renal pelvis.

**Figure 4.**
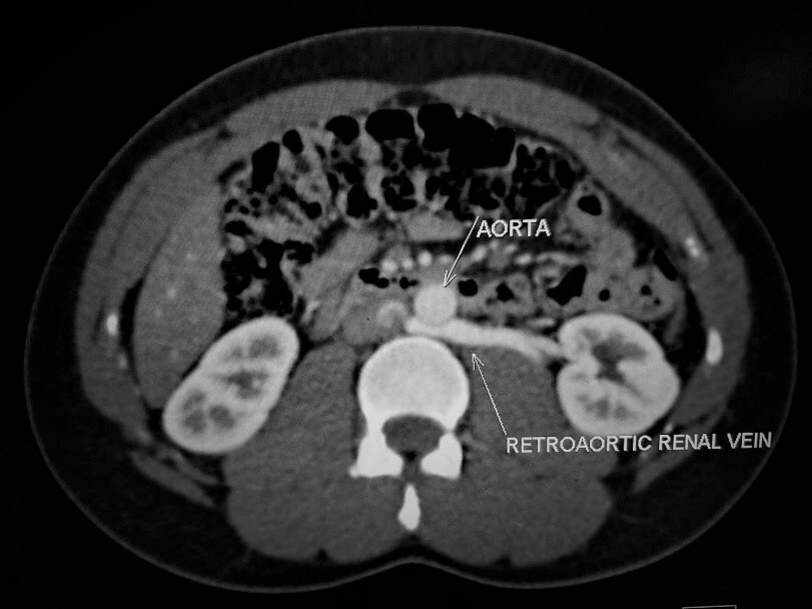
Axial reformat showing compression of the RLRV against the aorta.

The RLRV draining the lower pole was superior and posterior to the adjoining ILPRA. The vein had an intimate course with ILPRA (Figures 2 and 3). The diameter of the RLRV narrowed down to 0.29 cm between the aorta and the adjacent lumbar vertebrae compared to the hilar diameter of 1.1 cm (Figure 4). Distally the vein widened as it enters the IVC at the L3 level. The resultant of this compression gave rise to the diagnosis of posterior nut cracker phenomenon.

**Figure 5.**
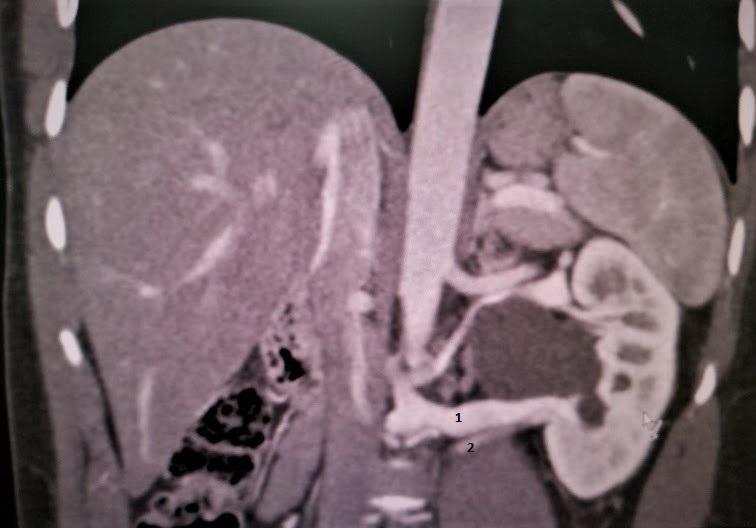
Coronal reformat showing the PUJ obstruction due to lower pole crossing vessels. Retroaortic Left renal vein (1) and Inferior pole Left renal artery (2).

**Figure 6.**
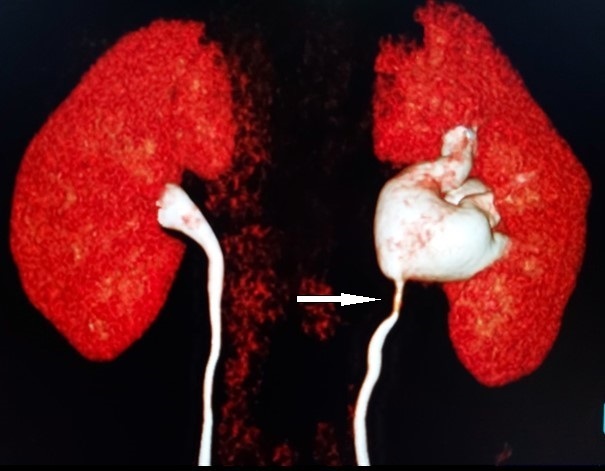
VRT delayed excretory image showing Left PUJ stenosis (white arrow).

There was dilation of the renal pelvis whose aetiology was attributed to the two lower pole anteriorly crossing vessels (ILPRA and RLRV) at the junction of left renal pelvis and the proximal ureter (Figure 5). A delayed excretory (limited scan area) was done 24 h later, which showed the dilatation of the renal pelvis and confirmed the stenosis at the pelviureteric junction (Figure 6).

## Discussion

The renovascular anatomy exhibits complex morphological variations in terms of their number, level of origin, diameter and topographical relationships. This case presents a rarity of simultaneous occurrence of arterial, venous anomalies and their associated vascular compression phenomena in a single patient. To the best knowledge of the author, no case report of this nature has been reported.

It is not uncommon to find variations in arterial, venous and ureteral anatomical patterning of the right and left kidneys. However, concomitant involvement with all three systems is a rarity.^1^ Due to their rarity, they can be easily missed on routine scans especially in patients with non-specific symptoms. Hence, comprehension of the typical imaging findings and associated clinical symptoms is critical particularly if surgical management is to be considered.^2^

These renovascular anomalies and compression phenomena may also occur in healthy individuals and it is vital to stratify the subset of patients who will benefit from surgical treatment.^2^ These is true in cases of renal transplantation, retroperitoneal surgery and other interventional procedures.^3^ Radiological diagnosis on variant renovascular anatomy may obviate untoward effects associated with surgical procedures such as laparoscopic surgery.^4^ The repair of renal vessels in laparoscopy is much more difficult compared to open surgery, often causing haemorrhage, a need for transfusion or conversion to laparotomy.^5^

## Renal artery anomalies

Normally, each kidney has a solitary renal artery.^6^ Approximately 70% of the population have a single renal artery arising from abdominal aorta.^7^ However, the remainder of the population have renal artery variations that differ in origin and number.^7^ The renal arteries may vary in their level of origin, caliber, obliquity, number and precise relation. The commonest site of origin of renal arteries from abdominal aorta is lateral (92%), and less commonly on anterolateral (6%) and posterolateral (2%) aspect of abdominal aorta at the level of L1-L2.^7^

Double renal arteries with an aortic origin are frequent vascular variations mainly due to persistence of the embryonic vessels and the lateral branches of the mesonephros during the embryological process.^6^

## Renal vein anomalies

The genesis of renal vein variants is as a result of complex embryological development of the inferior vena cava. There are three primitive cardinal veins (posterior cardinal, supracardinal and subcardinal) that appear, anastomose and regress in a sequential manner.^8^ The anastomotic communications between subcardinal and supracardinal channels form a collar of veins encircling the aorta.^9^ The ventral portion of the circumaortic collar persists as the normal left renal vein. If the dorsal portion of this collar persists, the left renal vein is posterior to the aorta forming a RLRV.^9^ The two most common variants are the circumaortic left renal vein (CLRV) and RLRV.^10^ Double renal veins are common unlike triple and quadruple renal veins that are very rarely reported in literature.^11^

Based on a meta-analysis, CLRV has been subclassified into three types. Type I CLRV with partial distal bifidity, in which the retroaortic branch receives the root of the hemiazygos; Type II CLRV with partial proximal bifidity(common), in which the origin is separated, and the two branches join together in anterior of the aorta; Type III (complete), comprising of two distinct thick venous trunks which remain separated until they join the IVC.^5^ Type III has two subtypes. Type III (inferior polar), in which the main vein, the superior one is preaortic, and the inferior polar vein is retroaortic. Type III (superior polar), in which the main trunk is horizontal, preaortic; it receives the adrenal and sometimes the gonadal gland.^5^ The superior polar vein is retroaortic, and usually has an oblique, inferior course towards the IVC.^5^ In this case report, this was a Type III (inferior polar) CLRV.

An accessory renal vein is considered a normal variant where the extra renal vein drains into inferior vena cava separately in addition to the renal vein proper.^11^They are more common on the right side due to the persistence of mesonephric veins and a rarity on the left because the largest component of the venous system regresses on that side.^11^

## Nutcracker phenomenon

Nutcracker phenomenon is classified as anterior nutcracker which is the compression of the left renal vein between abdominal aorta and superior mesenteric artery and posterior or pseudonutcracker or (rare) which is the compression of the retroaortic left renal vein between the abdominal aorta and vertebral column.^12^ Nutcracker phenomenon is also a rare cause of both microscopic and macroscopic haematuria and flank pain.^12^

According to a study by Kim et al., the beak angle of less 32 degrees has the highest diagnostic accuracy for anterior nut cracker phenomenon.^13^ Other less accurate diagnostic criteria are the ratio of LRV diameters at the hilar and aortomesenteric regions of more than 4.9 has a sensitivity of 66.7% and specificity of 100% for this condition.^14^ Compression ratios and contrast jetting phenomenon are also used.

However, unlike the anterior nut cracker phenomenon, the current studies have not based the diagnostic criteria of posterior nutcracker phenomenon on compression ratios or beak angle measurements. The posterior nutcracker phenomenon is diagnosed if there is decreased space between the aorta and the vertebra which results in compression of the RLRV.^9^ In this case report, there was narrowing of the RLRV between the abdominal aorta and the adjacent vertebrae (Figure 4).

Although uncommon, a radiological diagnosis is vital due to the high morbidity associated with the risk of secondary anaemia from haematuria, from long-term left renal vein hypertension, vascular thrombosis and even blood clots in the urinary system.^12^

## Vascular PUJ compression

Obstruction at the junction of the renal pelvis and the proximal ureter often manifests has hydronephrosis due to the impedance in the flow of urine.^15^ The cause PUJ obstruction can either be intrinsic or extrinsic. The commonest extrinsic cause in late childhood and adulthood are crossing vessels in close proximity to the ureter.^15^

These crossing vessels usually compress the ureteropelvic junction.^11^ A crossing vessel can be seen in 29–46% of cases of ureteropelvic junction obstruction.^11^ Commonly, the crossing vessel is an aberrant renal artery that crosses the pelviureteric junction anteriorly.^15^In this case report, the PUJ obstruction was as a result of two anteriorly crossing vessels (ILPRA and RLRV), which is a rarity (Figures 5 and 6).

Surgical intervention is required for patients with worsening renal function due to PUJ obstruction although open pyeloplasty had been the favoured method.^16^ However, minimally invasive endourologic techniques such endopyelotomy and laparoscopic or robot-assisted laparoscopic pyeloplasty have led to improved outcomes.^11^

Preoperative detection of crossing vessels at the PUJ, whether they are the primary cause of obstruction or an incidental finding, may play a critical role in surgical planning.^11^ This is due to the risk of haemorrhage during surgery. Studies show that the presence of crossing vessels reduces the success rate of antegrade endopyelotomy from 86 to 42%.^11^ In addition, crossing vessels are end arteries that lack anastomotic communication, which makes it vital to preserve them intraoperatively.^11^ This patient was scheduled for surgery hence the need for proper demonstration of the renal vasculature and identification of the cause of PUJ obstruction.

## Conclusion

Preoperative radiological diagnosis of renovascular variants and their associated vascular compression phenomena is vital particularly in patients with long-standing symptoms in whom surgery may be beneficial. In addition, it is also important for surgical planning thus reducing the risks associated with surgical management.

## Learning objectives

- Concomitant renovascular variants with associated vascular compression phenomena although rare can occur in a single patient.
- Optimization of imaging protocol (risk *vs* benefit) is essential for patients with renovascular anomalies and PUJ obstruction if they are scheduled for surgery.
- Delayed excretory scans will demonstrate the presence or absence of PUJ stenosis in relation to crossing vessels.

Permission to write the case report was sought and granted by the institution through the head of department subject to the author ensuring the anonymity and confidentiality of the patients under study.
